# Effects of exercise frequency on the gut microbiota in elderly individuals

**DOI:** 10.1002/mbo3.1053

**Published:** 2020-05-01

**Authors:** Qiwei Zhu, Shangfei Jiang, Guankui Du

**Affiliations:** ^1^ Department of Biochemistry and Molecular Biology Hainan Medical University Haikou China; ^2^ Human Anatomy Laboratory Hainan Medical University Haikou China

**Keywords:** elderly, exercise, gut microbiota, overweight

## Abstract

Growing evidence has shown that exercise can affect the gut microbiota. The effects of exercise frequency on the gut microbiota in elderly individuals are still largely unknown. In the present study, samples from 897 elderly and 1,589 adult individuals (18–60 years old) from the American Gut Project were screened. Microbial diversity and composition were analyzed by QIIME2, and microbial function was predicted by PICRUSt2. The outcomes were further analyzed by STAMP. The analysis showed that the α‐diversity of gut microbiota increased with increasing age, and regular exercise reshaped the alterations in microbial composition and function induced by aging. Moreover, the α‐diversity of gut microbiota was higher in overweight elderly individuals than in normoweight elderly individuals, and regular exercise significantly affected the microbial composition and function in overweight elderly individuals. In conclusion, we revealed that regular exercise benefits elderly individuals, especially overweight elderly individuals, by modulating the gut microbiota.

## Graphical abstract

This study investigated whether microbial diversity increases with increasing age. The gut microbiota composition of daily exercising elderly individuals approached that of adults (18–60 years old), and regular exercise resulted in an increased relative abundance of bacterial functional pathways. We also showed that overweight elderly individuals had increased microbial diversity and significant changes in the microbial composition.

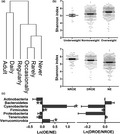

## 1 INTRODUCTION

In the intestine, hundreds of millions of bacteria play essential roles in host health (Bäckhed et al., 2004). The gut microbiota is composed of various microorganisms that form a complex ecological balance. The complexity and diversification of the gut microbiota benefit the host in many ways, such as by providing resistance against potential pathogens and increasing immunity (Round & Mazmanian, 2009). Metabolites produced by bacteria mediate host nutrition absorption and health. Thus, an imbalance in the gut microbiota could result in various disease pathogenicities, such as obesity and diabetes mellitus (Tremaroli & Bäckhed, 2012). The gut microbiota can be regulated by a range of factors, such as diet, antibiotic use, diseases, and exercise (Nicholson et al., 2012). Therefore, the gut microbiota is becoming a novel target for disease therapies.

Exercise is an important means to delay aging and prevent and manage diseases (Biddle & Batterham, 2015). Exercise can effectively reduce the risks of heart disease, stroke, hypertension, diabetes, cancer, and osteoporosis and improve depression (Biddle & Batterham, 2015). Exercise is beneficial to health by reducing body mass, the body mass index (BMI), the fat mass percentage, the fasting glucose level, and the fasting insulin level (Biddle & Batterham, 2015). Accumulating evidence shows that exercise affects the gut microbial composition, which might play a positive role in energy regulation (Mach & Fuster‐Botella, 2017; Monda et al., 2017). Animal studies have shown that exercise improves the diversity of the gut microbiome and changes the composition of bacteria in the gut (Brandt et al., 2018; Feng et al., 2017). Studies show that exercise could change the microbial composition at the phylum level, affecting the abundance of phyla such as Firmicutes, Proteobacteria, Prevotella, and Cyanobacteria (Lambert et al., 2015; Liu et al., 2015). The families Christensenellaceae and Coriobacteriaceae have shown increased abundance, suggesting a link between exercise and health improvement (Liu et al., 2015; Zhao et al., 2018). Furthermore, exercise training increases the production of short‐chain fatty acids (Feng et al., 2017). In humans, following an exercise challenge, the gut microbiota diversity and microbial composition are altered (Taniguchi et al., 2018; Whisner, Maldonado, Dente, Krajmalnik‐Brown, & Bruening, 2018). Similar results have been shown in athletes. Athletes have increased microbial diversity, which might be correlated with metabolic improvement and an inflammation reduction (Barton et al., 2018; Clarke et al., 2014). Thus, the exercise–gut microbiota axis might play an important role in maintaining health.

Overweight (BMI > 25) is becoming a global health problem. Exercise helps regulate body weight, as it is inversely associated with weight gain and contributes to weight loss (Jakicic, Rogers, Davis, & Collins, 2018). In high‐fat diet (HFD)‐induced obese mice, exercise counteracted the microbial imbalance, which was distinct from dietary effects, protected the intestinal barrier, and improved bile acid homeostasis (Carbajo‐Pescador et al., 2019; Evans et al., 2014). Moreover, genetic capacity studies predict that exercise increases tricarboxylic acid (TCA) cycle metabolism (Denou, Marcinko, Surette, Steinberg, & Schertzer, 2016). In humans, exercise‐induced alterations in microbial beta diversity depend on obesity status (Allen et al., 2018).

The gut microbiota undergoes aging‐related changes that may affect health. An early study showed that γ‐proteobacteria were enriched, while *Ruminococcus obeum* and its closely related phylotypes were hardly detected in elderly individuals (Hayashi, Sakamoto, Kitahara, & Benno, 2003). Studies have shown that the microbial community structure of elderly individuals is significantly different from that of young individuals (Claesson et al., 2011). Shen et al. (2018) found that the microbial changes caused by aging were characterized by reduced Bacteroidetes abundance. Ogawa, T., et al. suggested that adequate exercise was important for maintaining health because it manipulates the gut microbiota in elderly individuals (Shimizu, 2018). However, the effects of exercise frequency on the gut microbiota of elderly individuals remain largely unknown. The present study was carried out to characterize regular exercise‐induced changes in the properties of the gut microbiota in elderly individuals and to further analyze regular exercise‐induced changes that benefit overweight elderly (OE) individuals.

## 2 METHODS

### 2.1 Data sources

The data for this study were obtained from the American Gut Project (AGP) (McDonald et al., 2018). This project was the largest crowd‐funded research project created by Rob Knight and Jeff Leach. Samples taken from participants were kept at the Rob Knight Laboratory at the University of California, San Diego. DNA was extracted from the samples and then sequenced using an Illumina MiSeq sequencer.

The sample data were obtained from the Sequence Read Archive (SRA) (https://www.ncbi.nlm.nih.gov/sra), in which 25,376 samples from the American Gut Project were deposited, including 19,988 samples collected from fecal specimens. Upon excluding the samples without basic information (sex, age, BMI, or self‐reported exercise frequency), from patients who received antibiotic treatment within 6 months, from patients with tumors, and from patients who went on a trip within the previous three months, the data of 3,795 samples remained. Of these, samples from 897 elderly individuals and 1,589 18‐ to 60‐year‐old adults (adults_18‐60_) with normoweight were included in the study.

### 2.2 Experimental design

To determine the alterations in gut microbiota with age, 1,589 samples from adults_18‐60_ and 462 samples from elderly individuals with a normal BMI were divided into 6 groups according to age (approximately ten years per age group): the 18‐ to 30‐year‐old group (329 individuals), 31‐ to 40‐year‐old group (433 individuals), 41‐ to 50‐year‐old group (428 individuals), 51‐ to 60‐year‐old group (399 individuals), 61‐ to 70‐year‐old group (371 individuals), and over 70‐year‐old group (91 individuals; Table 1).

**TABLE 1:** Number of samples used in different groups

Adults_18−60_ (*n* = 1,589)	Normoweight	Overweight	Underweight
18‐ to 30‐year‐old	329		
31‐ to 40‐year‐old	433		
41‐ to 50‐year‐old	428		
51‐ to 60‐year‐old	399		

Elderly (*n* = 897)	Normoweight	Overweight	Underweight
	462	413	22
61‐ to 70‐year‐old	371		
over 70‐year‐old	91		

Exercise frequency in elderly (*n* = 897)		Exercise in overweight elderly (*n* = 413)	
Daily exercise	194	Daily or regular exercise	74
Regular exercise	360	Never or rare exercise	222
Occasional exercise	209		
Rare exercise	102		
Never exercise	32		

To define the benefit of exercise in elderly individuals, the 897 elderly individual samples were divided into the daily exercise group (194 individuals), regular exercise group (360 individuals), occasional exercise group (209 individuals), rare exercise group (102 individuals), and never exercise group (32 individuals; Table 1).

To determine the alterations in gut microbiota with BMI in elderly individuals, elderly individual samples were further divided into 3 groups according to BMI: the underweight elderly group (BMI < 18.5) (22 elderly individuals), normoweight elderly (NE) group (18.5 < BMI<25) (462 elderly individuals), and overweight elderly (OE) group (BMI > 25) (413 elderly individuals; Table 1).

To define the benefit of exercise in OE individuals, the OE group was further divided into the daily or regular exercise in OE individuals (DROE) group (74 elderly individuals) and the never or rare exercise in OE individuals (NROE) group (222 elderly individuals). OE individuals who occasionally exercised (117) were excluded from the analysis (Table 1).

### 2.3 FASTQ format conversion

The SRA files were downloaded to a computer, and the sratoolkit tools were used to convert the SRA data into the fastq format. The fastq‐dump.exe command was used to convert the format.

### 2.4 Data processing

QIIME version 2 was utilized to process the data (Brandt et al., 2018). First, the Deblur plugin was used for sequence quality control. The operational taxonomic units (OTUs) resulting from Deblur in QIIME2 were created by grouping unique sequences that had the equivalent of 100% similarity to OTUs in QIIME1 (Brandt et al., 2018). The feature‐table‐table seqs command mapped feature IDs to sequences. The QIIME diversity alpha‐rarefaction visualizer was utilized to explore alpha diversity. Next, the taxonomic composition of the samples was explored. The naive Bayes classifier and the q2‐feature‐classifier plugin with the Greengenes 13.8 database were used to assign taxonomy to the sequences and map the sequences. The relative abundances at the phylum and family levels were determined based on OTU tables.

PICRUSt2 was used to predict metagenomic functions based on the normalized OTU tables (Douglas et al., 2020).

### 2.5 Statistical analysis

STAMP was used to calculate the level of significance (Parks, Tyson, Hugenholtz, & Beiko, 2014). Welch's *t* tests (two‐sided) were used for two‐group comparisons. Welch's inverted confidence interval (CI) method was used for CI calculation. The Benjamini–Hochberg false discovery rate method was used to calculate adjusted *p*‐values.

### 2.6 Patient and public involvement statement

Patients were not included in the sampling for this study.

## 3 RESULTS

### 3.1 Microbial composition was altered by age

**FIGURE 1:**
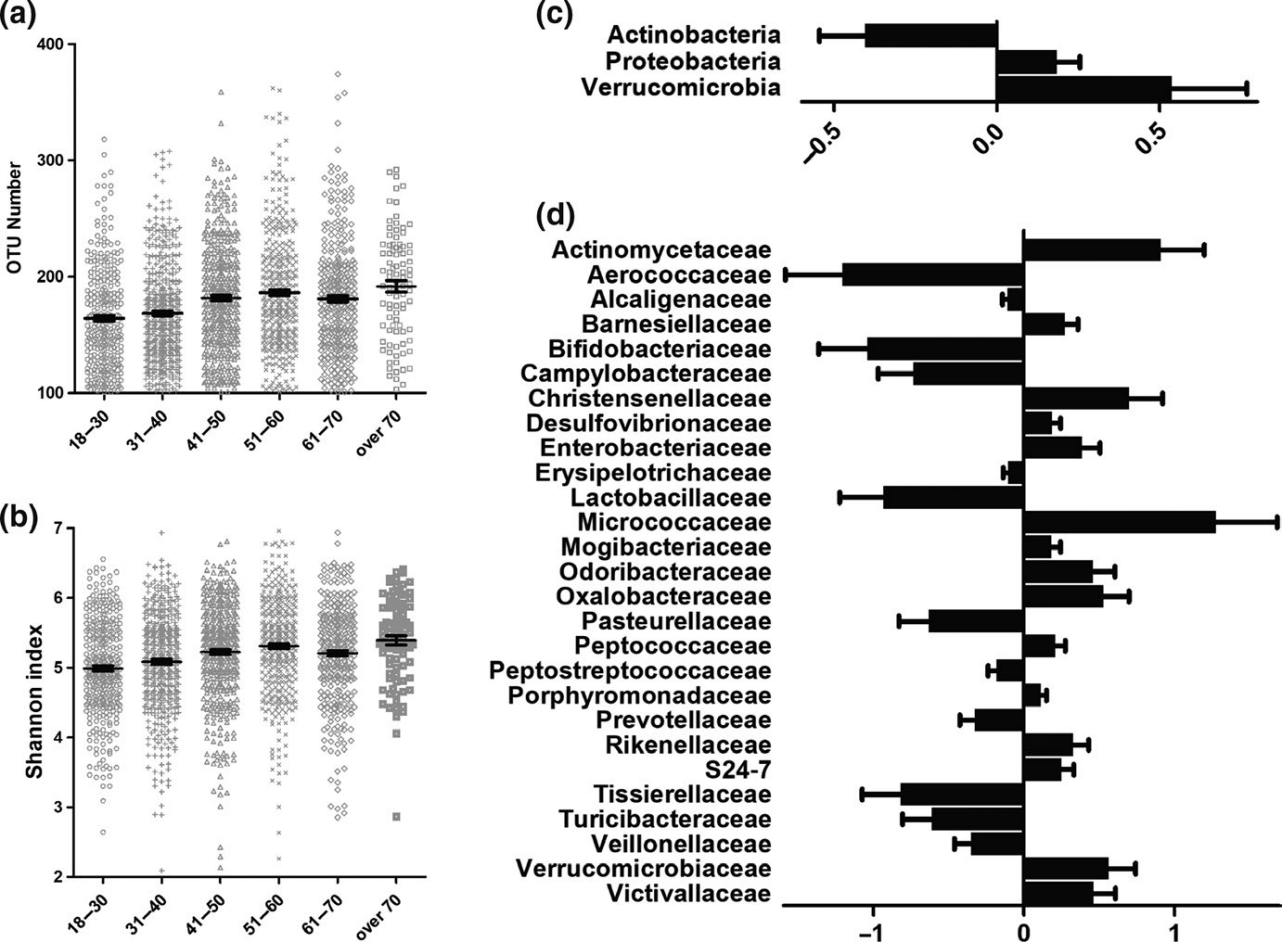
Alteration of the microbial composition in elderly individuals. Both (a) OTU number and (b) Shannon index showed that the microbial α‐diversity increased with age in 2,011 people with a normal BMI. Comparing the microbial composition in elderly individuals to that in adults_18‐60_, (c) three phyla were significantly changed. (d) Twenty‐seven families were significantly changed in the microbial composition in elderly individuals with different exercise frequencies compared with that in adults_18‐60_

Of the 25,376 samples screened by the American Gut Project, samples from 897 elderly individual samples and 1,589 adults_18‐60_ (18–60 years old) with a normal BMI (18.5 to 25) were analyzed in this study. The α‐diversity analysis showed that the OTU numbers were 164.3, 168.6, 181.6, 186.3, 181.0, and 191.7 (*p* < .0001), while the Shannon indices were 4.989, 5.083, 5.222, 5.309, 5.203, and 5.391 (*p* < .0001) in the 18‐ to 30‐year‐old group, 31‐ to 40‐year‐old group, 41‐ to 50‐year‐old group, 51‐ to 60‐year‐old group, 61‐ to 70‐year‐old group, and over the 70‐year‐old group, respectively (Figure 1a,b). Thus, the microbial α‐diversity significantly increased with age.

Since α‐diversity significantly changed with age, we detected the gut microbiota composition in elderly individuals and compared it with that of adults_18‐60_ (Figure 1c,d). At the phylum level, the relative abundances of Proteobacteria and Verrucomicrobia were significantly increased by 20.3% (*p* < .05) and 71.1% (*p* < .05), respectively, while the relative abundance of Actinobacteria was significantly decreased by 33.3% (*p* < .05) in the elderly individuals compared with the adults_18‐60_. At the family level, the relative abundances of Aerococcaceae, Bifidobacteriaceae, Lactobacillaceae, Pasteurellaceae, Prevotellaceae, Tissierellaceae, Turicibacteraceae, and Veillonellaceae were significantly increased by 70.1%, 64.6%, 60.6%, 46.8%, 27.7%, 55.8%, 45.8%, and 29.6%, respectively (*p* < .05), while the relative abundances of Barnesiellaceae, Christensenellaceae, Desulfovibrionaceae, Enterobacteriaceae, Odoribacteraceae, Oxalobacteraceae, Rikenellaceae, and Verrucomicrobiaceae were significantly decreased by 31.3%, 101.5%, 20.4%, 46.8%, 58.5%, 70.2%, 38.7%, and 75.8%, respectively (*p* < .05), in the elderly individuals compared with the adults_18‐60_.

### 3.2 Microbial composition and function were altered by exercise frequency

**FIGURE 2:**
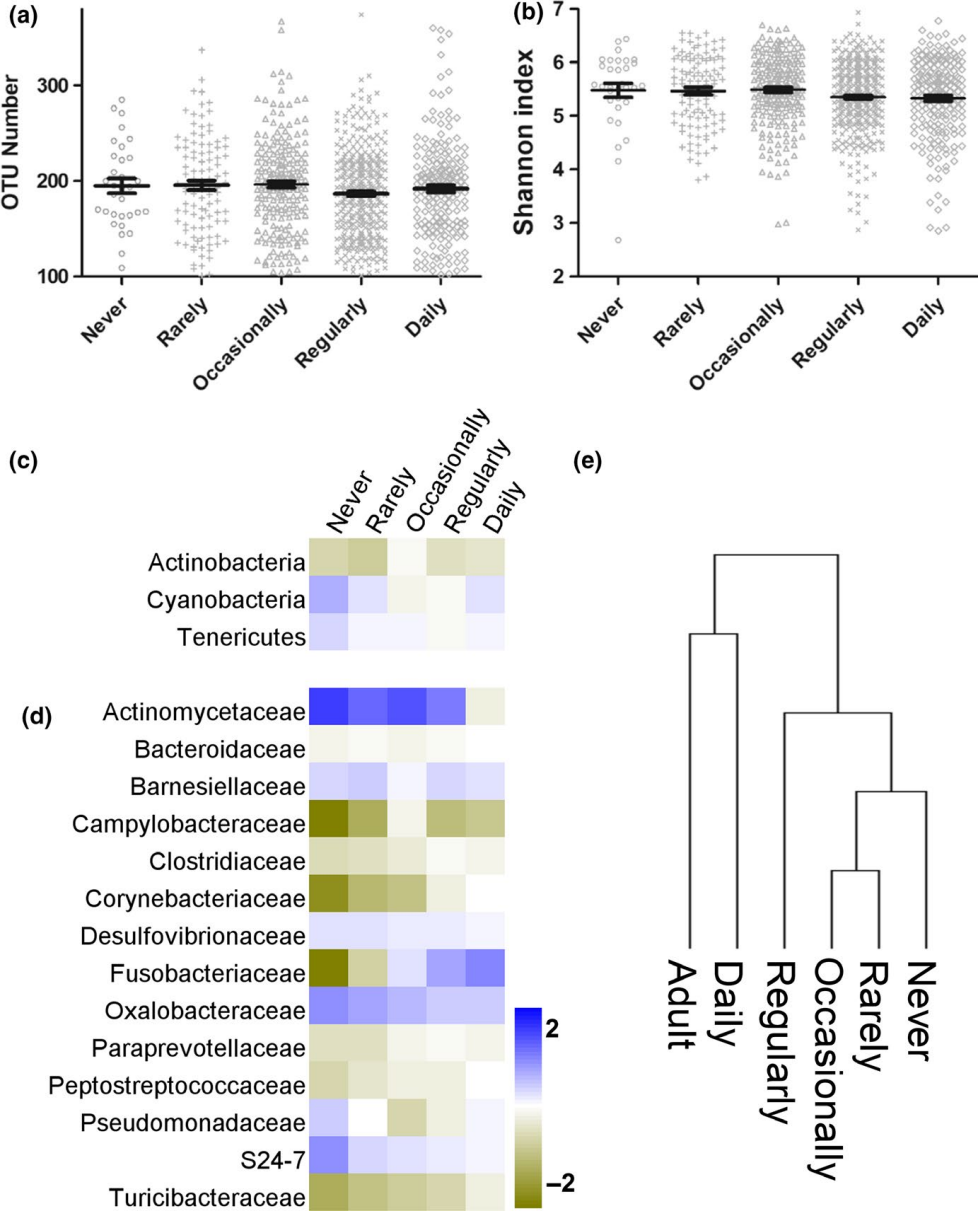
Alteration of microbial function in the DRE group. Both (a) OTU number and (b) Shannon index showed that exercise frequency affected α‐diversity in elderly individuals. Exercise‐induced 3 phyla (c) and 14 families (d) to gradually approach the levels of those in adults_18‐60_. (e) Evolutionary analysis of the microbial composition at the family level

To determine the effect of exercise frequency on microbial α‐diversity in elderly individuals, elderly individual samples were divided into 5 groups (never, rare (a few times/month), occasional (1–2 times/week), regular (3–5 times/week), and daily). The analysis showed that the OTU numbers were 194.9, 195.5, 196.3, 186.7, and 191.9 (*p* > .05), while the Shannon indices were 5.478, 5.466, 5.489, 5.351, and 5.332, respectively (*p* > .05; Figure 2a,b). Thus, these results suggested that the microbial α‐diversity was almost unaffected by exercise frequency in elderly individuals.

To define the benefit of exercise in elderly individuals, the microbial abundances of the never, rare, occasional, regular, and daily exercise groups were compared with that of adults_18‐60_ (Figure 2c,d). At the phylum level, the relative abundances of Actinobacteria, Cyanobacteria, and Tenericutes in elderly individuals gradually approached those in adults_18‐60_. At the family level, the relative abundances of Actinomycetaceae, Desulfovibrionaceae, S24‐7, Pseudomonadaceae, Barnesiellaceae, and Oxalobacteraceae gradually decreased with exercise frequency. The relative abundances of Campylobacteraceae, Fusobacteriaceae, Turicibacteraceae, Paraprevotellaceae, Clostridiaceae, Peptostreptococcaceae, Corynebacteriaceae, and Bacteroidaceae gradually increased with exercise frequency. As shown in Figure 2e, the evolutionary analysis revealed that the abundances in the daily exercise group were the closest to those in the adults_18‐60_ at both the phylum and family levels.

**FIGURE 3:**
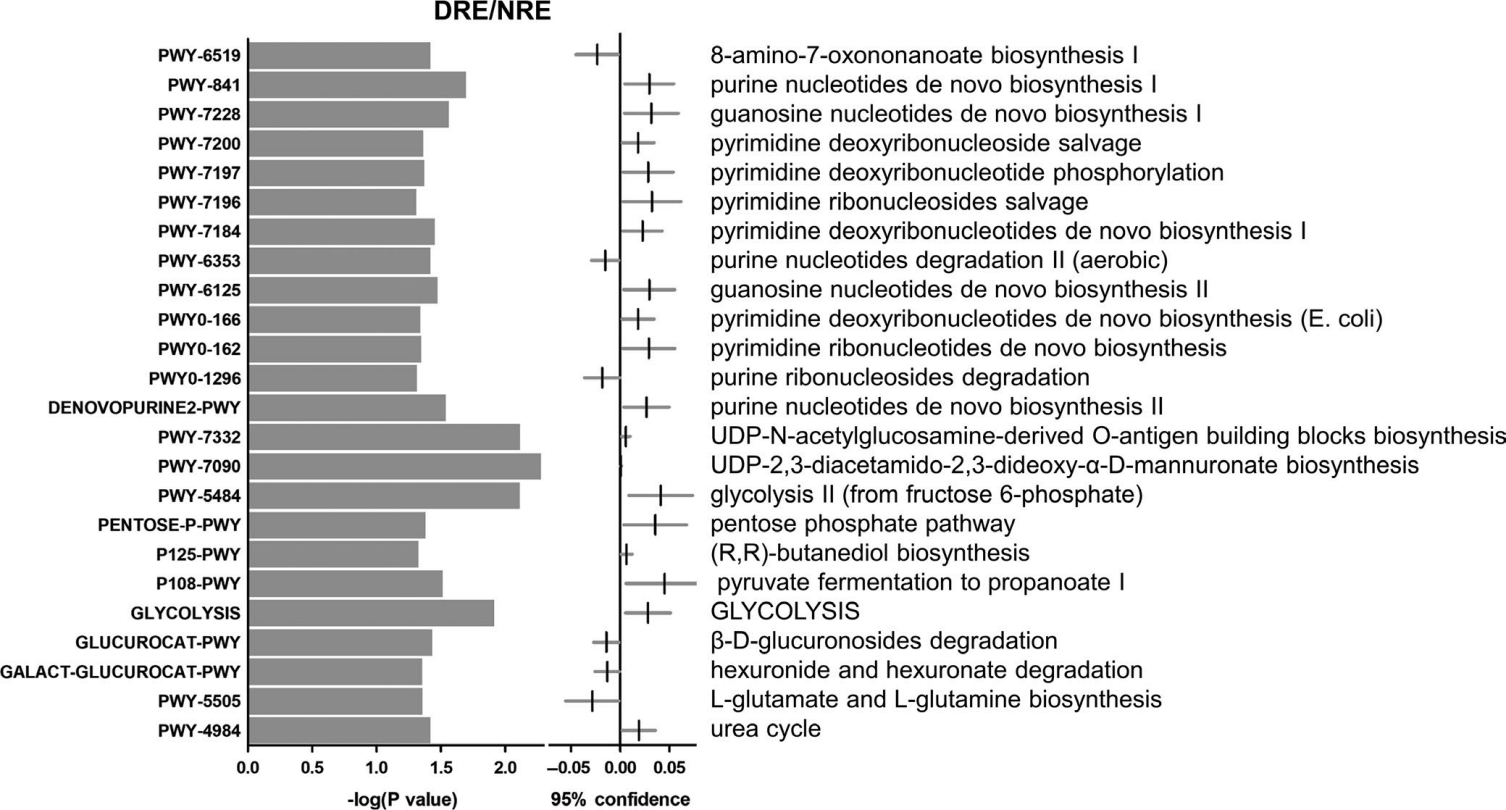
Alteration of microbial function in the DRE group. Twenty‐nine pathways were significantly changed in the DROE group compared with the NROE group. The 29 pathways were associated with vitamin, nucleotide, glucose, and amino acid metabolism

For analysis of microbial function, elderly individual samples were recombined into the daily or regular exercise (DRE) group and never or rare exercise (NRE) group (Figure 3). Twenty‐four pathways were identified in the comparison of the DRE and NRE groups (*p* < .05), including 1 vitamin‐related pathway (8‐amino‐7‐oxononanoate biosynthesis I), 12 nucleotide metabolism‐related pathways (purine nucleotides de novo biosynthesis I, guanosine nucleotides de novo biosynthesis I, pyrimidine deoxyribonucleoside salvage, pyrimidine deoxyribonucleotide phosphorylation, pyrimidine ribonucleosides salvage, pyrimidine deoxyribonucleotides de novo biosynthesis I, purine nucleotides degradation II (aerobic), guanosine nucleotides de novo biosynthesis II, pyrimidine deoxyribonucleotides de novo biosynthesis (E. coli), pyrimidine ribonucleotides de novo biosynthesis, purine ribonucleosides degradation, and purine nucleotides de novo biosynthesis II), 2 cell wall biosynthesis‐related pathways (UDP‐N‐acetylglucosamine‐derived O‐antigen building blocks biosynthesis and UDP‐2,3‐diacetamido‐2,3‐dideoxy‐α‐D‐mannuronate biosynthesis), 7 glucose metabolism‐related pathways (glycolysis II (from fructose 6‐phosphate), pentose phosphate pathway, (R,R)‐butanediol biosynthesis, pyruvate fermentation to propanoate I, glycolysis, β‐D‐glucuronosides degradation, and hexuronide and hexuronate degradation), and 2 amino acid metabolism‐related pathways (l‐glutamate and l‐glutamine biosynthesis and urea cycle). The relative abundances of 18 of those pathways were significantly higher, while the abundances of 5 of those pathways were significantly lower in the DRE group than in the NRE group. Therefore, these results suggest that regular exercise significantly modulated microbial function in elderly individuals.

### 3.3 Microbial α‐diversity was altered by regular exercise in OE individuals

**FIGURE 4:**
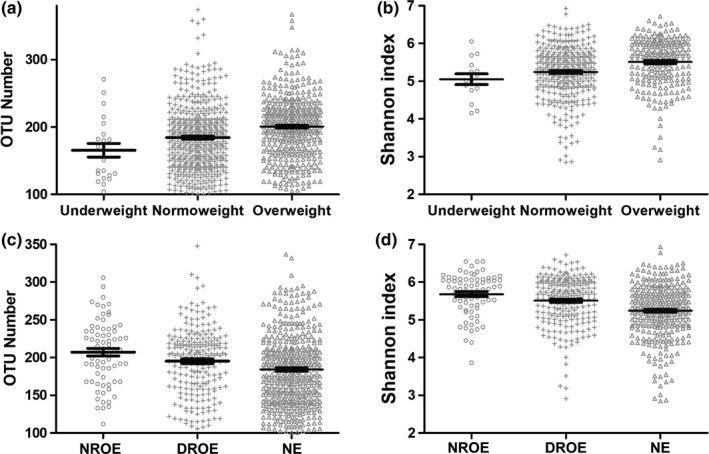
Alteration of the microbial α‐diversity in OE individuals. Both (a) OTU number and (b) Shannon index showed that the microbial α‐diversity increased with BMI. Both (c) OTU number and (d) Shannon index showed that the DROE group had altered microbial α‐diversity

The analysis showed that the OTU numbers were 165.5, 184.3, and 200.6 (*p* < .001), while the Shannon indices were 5.054, 5.244, and 5.508 (*p* < .001) in the underweight elderly, NE, and OE groups, respectively (Figure 4a,b). Thus, microbial α‐diversity significantly increased with BMI.

The α‐diversity analysis showed that the OTU numbers were 207.2 and 195.2 (*p* < .001), while the Shannon indices were 5.681 and 5.508 (*p* < .001) in the DROE and NROE groups, respectively (Figure 4c,d). Thus, the microbial α‐diversity was significantly affected by exercise in OE individuals.

### 3.4 Microbial abundance was partially restored by regular exercise in OE individuals

**FIGURE 5:**
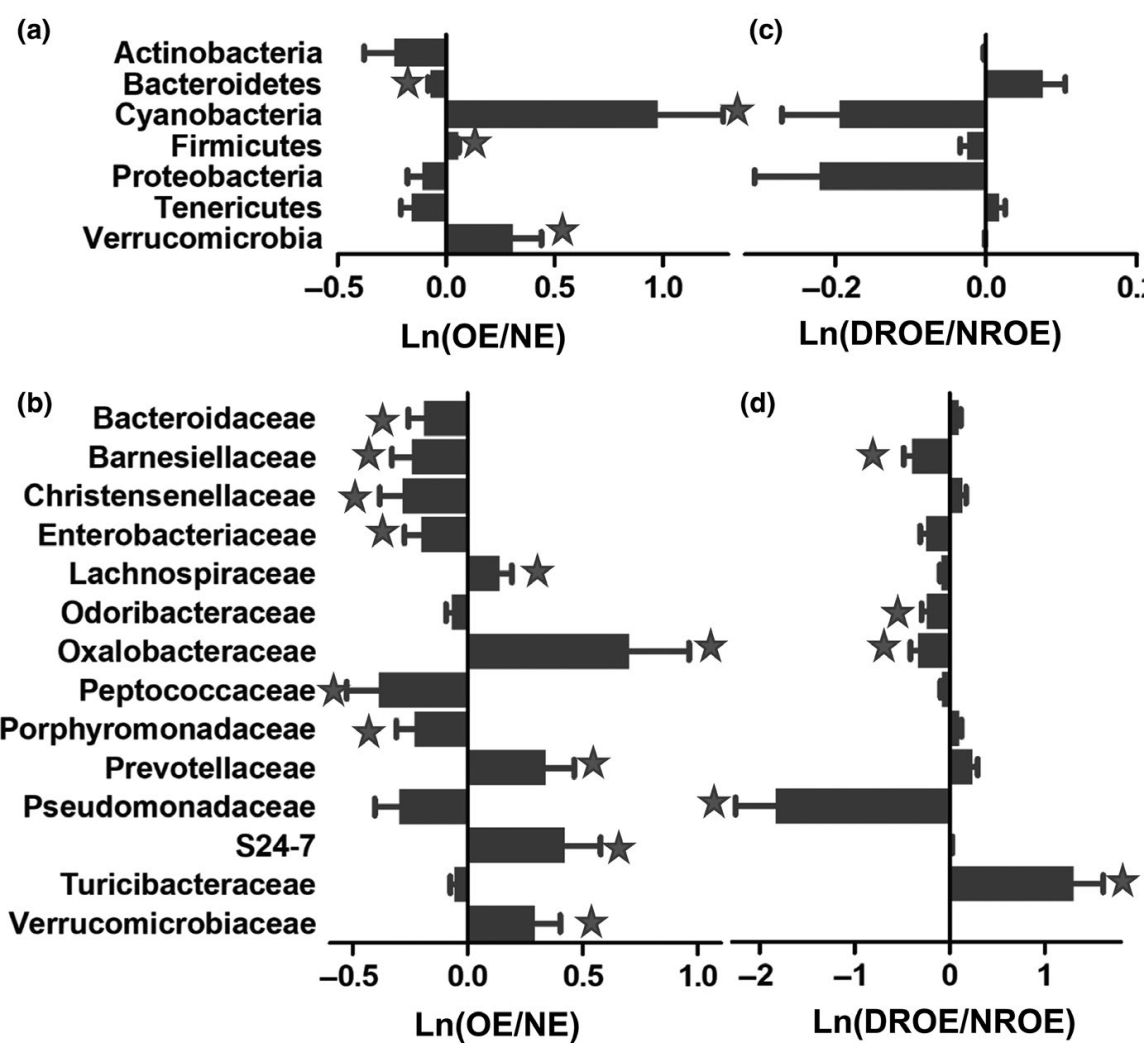
Alteration of the microbial composition in OE individuals. Comparison of the microbial compositions in OE and NE individuals at the (a) phylum and (b) family levels. Comparison of the microbial composition in the DROE and NROE groups at the (c) phylum and (d) family levels. The star represents *p* < .05

Next, the abundances of gut microbiota constituents were detected at the phylum and family levels in OE individuals (Figure 5). At the phylum level, the relative abundances of Cyanobacteria, Firmicutes, and Verrucomicrobia were increased by 165.7%, 6.1%, and 36.3% (*p* < .05), respectively, while the Bacteroidetes abundance was decreased by 6.7% in OE individual samples (*p* < .05) compared with (NE) individual samples. At the family level, the relative abundances of Verrucomicrobiaceae, S24‐7, Prevotellaceae, Oxalobacteraceae, and Lachnospiraceae were increased by 34.2%, 52.7%, 40.4%, 102.4%, and 15.1%, respectively, while the relative abundances of Enterobacteriaceae, Christensenellaceae, Barnesiellaceae, Bacteroidaceae, Porphyromonadaceae, and Peptococcaceae were decreased by 18.1%, 24.3%, 21.4%, 17.2%, 20.3%, and 31.9%, respectively, in the OE individuals (*p* < .05) compared with the NE individuals.

As shown in Figure 5, exercise affected the gut microbial composition in OE individuals. At the phylum level, the relative abundances of Bacteroidetes, Cyanobacteria, Firmicutes, Tenericutes, and Verrucomicrobia were partially restored in exercising (*p* > .05) compared with nonexercising OE individual samples. At the family level, the relative abundance of Turicibacteraceae was significantly increased, while the relative abundances of Pseudomonadaceae, Oxalobacteraceae, Odoribacteraceae, and Barnesiellaceae were significantly decreased in the frequently exercising OE individuals compared with nonexercising OE individuals.

### 3.5 Microbial functions were partially restored by regular exercise in OE individuals

Microbial functions were detected in OE individuals, and it was found that the relative abundances of 129 pathways were significantly changed (Appendix 1: Table A1). The relative abundances of 79 pathways were significantly increased, while those of 50 pathways were significantly decreased in the overweight group compared with the normoweight groups. Moreover, 25 pathways were identified by comparing the DROE and NROE groups (Appendix 2: Table A2). Among them, 19 pathways were significantly higher and 6 pathways were significantly lower in the DROE group than in the NROE group.

**FIGURE 6:**
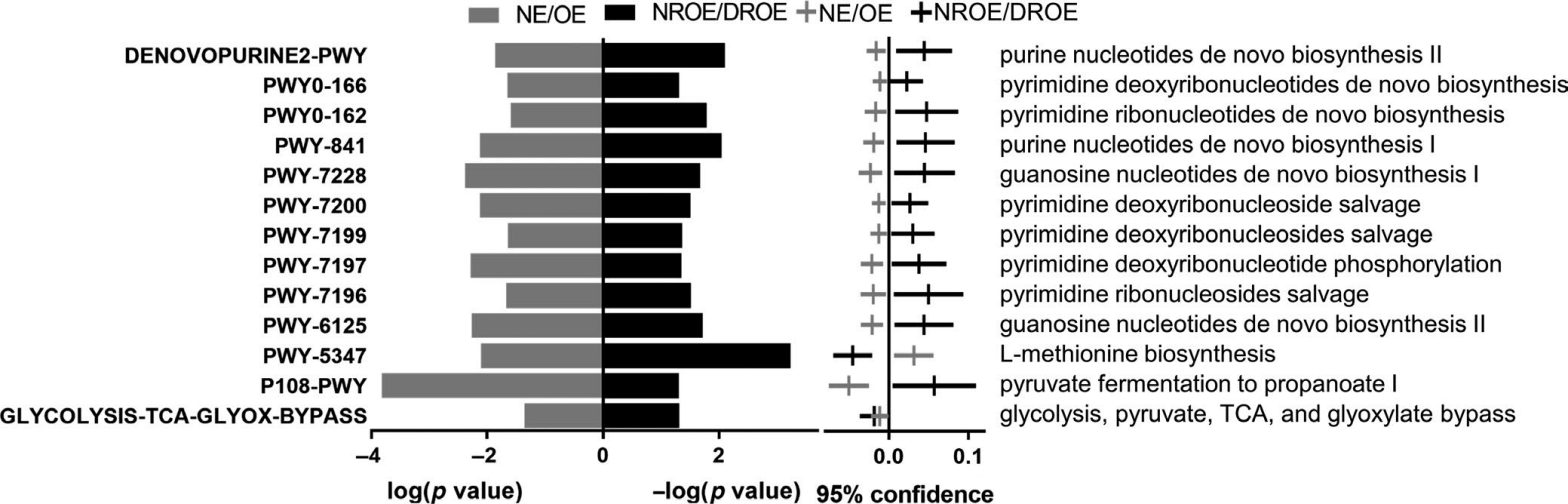
Alteration of microbial function in OE individuals. Thirteen pathways showed opposite trends in the NE/OE and DROE/NROE groups

Notably, 13 common pathways were identified (Figure 6). Among them, 12 pathways that were changed by overweight were restored by frequent exercise. The abundances of purine nucleotides de novo biosynthesis II, pyrimidine deoxyribonucleotides de novo biosynthesis, pyrimidine ribonucleotides de novo biosynthesis purine nucleotides de novo biosynthesis I, guanosine nucleotides de novo biosynthesis I, pyrimidine deoxyribonucleoside salvage, pyrimidine deoxyribonucleosides salvage, pyrimidine deoxyribonucleotide phosphorylation, pyrimidine ribonucleosides salvage, guanosine nucleotides de novo biosynthesis II, and pyruvate fermentation to propanoate I were significantly increased in OE individuals and significantly decreased in those in the frequent exercise group. The abundance of l‐methionine biosynthesis was significantly decreased in OE individuals and significantly increased in the frequent exercise group.

## 4 DISCUSSION

The gut microbiota plays a major role in age‐related diseases. Moreover, changes in gut microbiota diversity have profound impacts on host metabolism. In the present study, we detected the effect of exercise on the gut microbiota in elderly individuals by using data obtained from the American Gut Project. The microbial α‐diversity increased with age in the normal BMI population, and the gut microbiota was restored by exercise in elderly individuals.

A recent study showed that microbial α‐diversity is positively associated with age in populations such as the United States, the United Kingdom, and Colombia (de la Cuesta‐Zuluaga et al., 2019). Kasai et al. (2015) showed that bacterial diversity was significantly higher in obese individuals than in nonobese individuals in a Japanese population. The Shannon index was increased in those with an obese BMI in a randomly selected Alabama resident study (Davis, Yadav, Barrow, & Robertson, 2017). Infants who were obese at 6 months of age had higher levels of alpha diversity than nonobese infants (Ville, Levine, Zhi, Lararia, & Wojcicki, 2020). However, some studies showed that obesity/overweight was associated with decreased α‐diversity in animals or humans (Chen et al., 2020; Da Silva, Monteil, & Davis, 2020; van der Merwe et al., 2020). Moreover, a recent study showed no differences in the α‐diversity in obese individuals in an Asian population (Koo et al., 2019). In this study, we analyzed the association of gut microbial α‐diversity with age and BMI. The analysis showed that the microbial α‐diversity increased with increasing age. In addition, the microbial α‐diversity increased with increasing BMI in elderly individuals. We also showed that the microbial α‐diversity was decreased in the overweight group of whole AGP samples (data not shown). Therefore, the analysis results based on AGP are in accordance with the finding that microbial α‐diversity is positively associated with increased age and obesity in elderly individuals.

In humans, the effect of exercise on gut microbial diversity is somewhat controversial. Studies have shown that exercise can increase microbial α‐diversity (Houghton et al., 2018; Keohane et al., 2019). However, other studies have shown that exercise dOEs not affect the α‐diversity of the gut microbiota (Cronin et al., 2018; Kern et al., 2020; Kim & Kang, 2019). Besides, studies have shown that exercise might be negatively associated with microbial α‐diversity (Allen et al., 2015; Mika et al., 2015). The reason for the discrepancies may be that the previous studies were small in scale. In the present study, we found that the microbial α‐diversity decreased with increasing exercise frequency in elderly individuals, and the microbial α‐diversity decreased in the OE group. Therefore, these results seem to be consistent with other research that found a negative association with exercise frequency in elderly individuals, especially in OE individuals.

Comparing the microbial composition of elderly individuals with that of adults_18‐60_, we found that 3 phyla and 27 families had significantly different abundances. At the phylum level, the abundance of Proteobacteria was significantly increased, while that of Actinobacteria was significantly decreased. The Bacteroidetes abundance was lower in the elderly individual group (37.7%) than in the adults_18‐60_ (39.0%; *p* > .05), while the Bacteroidetes/Firmicutes ratio was not significantly different between the two groups. At the family level, the abundances of Desulfovibrionaceae and Enterobacteriaceae were significantly increased, while that of Bifidobacteriaceae was significantly decreased in elderly individuals. Actinobacteria species are key in maintaining gut homeostasis and play an important role in the treatment of gastrointestinal diseases and systemic diseases (Jami, Ghanbari, Kneifel, & Domig, 2015). Bifidobacteria species (phylum: Actinobacteria) are widely used as probiotics and have demonstrated beneficial effects under a variety of pathological conditions (Shen et al., 2018). Studies have indicated that Proteobacteria may be a characteristic microorganism of diseases, including metabolic disorders and inflammatory bowel disease. Proteobacteria can cause inflammation and lead to disease development (Rizzatti, Lopetuso, Gibiino, Binda, & Gasbarrini, 2017). The increase in intestinal Desulfovibrionaceae abundance is an important feature of colitis (Leonardi et al., 2017). Enterobacteriaceae are important pathogens because of their capacity to produce endotoxins (Xie et al., 2017). Therefore, our results suggested that aging induced an increase in harmful bacteria and a decrease in beneficial microbes.

The microbial function analysis predicted that 25 pathways were significantly different between elderly individuals and adults_18‐60_ (Appendix 2: Table A2). The amino acid metabolism‐related pathways were altered, which might result in decreased biosynthesis of essential amino acids (threonine, phenylalanine, lysine, and tryptophan) and aromatic amino acids (tyrosine, phenylalanine, and tryptophan). Vitamin biosynthesis‐related pathway abundances were decreased. A previous study showed that the gut microbiota was involved in essential amino acid homeostasis (Lin, Liu, Piao, & Zhu, 2017). The gut microbiota can synthesize K and B vitamins, including biotin, riboflavin, cobalamin, folic acid, nicotinic acid, pantothenic acid, pyridoxine, and thiamine (Rowland et al., 2018). It is estimated that vitamins synthesized by gut microbes can provide more than a quarter of the intake (Rowland et al., 2018). Therefore, our results suggest that age induced a decrease in the production of essential amino acids and vitamins by the gut microbiota, thereby reducing these substances in elderly individuals.

Studies have shown that exercise is linked to microbial composition (Murtaza et al., 2019; Petersen et al., 2017; Scheiman et al., 2019). Murtaza et al. (2019) identified two dominant enterotypes (Bacteroides and Prevotella) in elite endurance athletes. Scheiman et al. (2019) showed that Veillonella was linked to exercise performance. Petersen et al suggested that Prevotella was correlated with exercise (Petersen et al., 2017). Barton et al found enrichment of Akkermansia in athletes (Barton et al., 2018). We compared the microbial composition of each exercise frequency group with that of adults_18‐60_ and found that the abundances of 3 phyla and 14 families gradually approached those in adults_18‐60_ with an increase in exercise frequency. The abundance of Actinobacteria gradually increased and was similar to that in adults_18‐60_. Additionally, the abundance of Cyanobacteria gradually decreased and approached that in adults_18‐60_. Cyanobacteria are associated with diseases and, in some cases, with human and animal death (Lange et al., 2018). Cyanobacteria release their toxins, such as lipopolysaccharides, in B cells in the gut (Swanson‐Mungerson et al., 2017). Furthermore, the evolutionary analysis showed that the microbial composition in the daily exercise group was closest to that in adults_18‐60_. We also revealed that daily exercise shifts the gut microbiota to a younger phenotype.

In OE individuals, the abundances of Actinobacteria and Bacteroidetes were decreased, while the abundances of Cyanobacteria and Firmicutes were increased at the phylum level. The Bacteroidetes/Firmicutes ratio was significantly decreased. At the family level, the abundances of S24‐7 and Lachnospiraceae were increased, while the abundances of Christensenellaceae, Barnesiellaceae, and Bacteroidaceae were decreased in OE individuals. It was reported that S24‐7 enrichment was associated with a high‐fat diet in diabetes‐sensitive mice (Serino et al., 2012). Kameyama et al showed that intestinal colonization by a Lachnospiraceae bacterium promoted the development of diabetes in obese mice (Kameyama & Itoh, 2014). It was reported that Christensenellaceae bacteria were beneficial for human health and had an increased abundance in lean people (Requena, Martínez‐Cuesta, & Peláez, 2018). Barnesiellaceae could serve as a marker to discriminate lean and obese individuals (Rodriguez, Benninghoff, Aardema, Phatak, & Hintze, 2019). Hakkak, Korourian, Foley, & Erickson (2017) showed that lean rats exhibited much lower Firmicutes to Bacteroidetes ratios than obese rats. A recent report showed that Bacteroidaceae might play a role in bamboo shoot fiber‐mediated suppression of high‐fat diet‐induced obesity (Li, Guo, Ji, & Zhang, 2016). Therefore, our results suggest an increase in harmful bacteria and a decrease in beneficial microbes in OE individuals.

Next, we revealed that the microbial α‐diversity was significantly decreased, while the microbial abundance approached the mean level in elderly individuals in the DROE group. At the phylum level, the abundance of Bacteroidetes was increased, while the abundances of Proteobacteria, Cyanobacteria, and Firmicutes were decreased in the DROE group compared with the NROE group. At the family level, the abundance of Lachnospiraceae was decreased, while the abundances of Christensenellaceae and Bacteroidaceae were increased in OE individuals. Exercise effectively counteracted obesity‐induced microbial imbalances, increased the abundance of the Bacteroides genera, and decreased the abundance of the Porphyromonas genera in male Wistar rats (Carbajo‐Pescador et al., 2019). A 6‐week endurance exercise regimen in overweight women revealed that training slightly increased genus‐level β‐diversity and decreased Proteobacteria abundance (Munukka et al., 2018). Therefore, our results suggest that regular exercise might play a role in decreasing harmful bacteria and increasing beneficial microbes in OE individuals.

Thirteen common pathways were isolated and showed opposite trends in the OE/NE and DROE/NROE groups**.** Ten pathways related to nucleotide biosynthesis were significantly inhibited in OE individuals and enhanced after regular exercise. Thus, these results suggest that regular exercise might modulate nucleotide biosynthesis, which is important for microorganism growth.

This study relied on the participants' accurate self‐reporting of the frequency of exercise without tracking the intensity of exercise. Studies have shown that insufficient or excessive exercise intensity has a significant impact on the structure and function of the intestinal flora. Moreover, the main research participants in this study were Caucasian, and the amount of data from other ethnic groups is small. Future research should specifically study other ethnic groups for comparative analysis.

## 5 CONCLUSION

In conclusion, our results revealed that microbial diversity increased with increasing age. The gut microbiota composition of daily exercising elderly individuals approached that of adults_18‐60_, and regular exercise resulted in an increased relative abundance of bacterial functional pathways related to nucleotide metabolism, glucose metabolism, and lipid metabolism. We also showed that OE individuals had increased microbial diversity and significant changes in the microbial composition, which responded with alterations in bacterial functional pathways related to vitamin, nucleotide, and glucose metabolism. Furthermore, regular exercise partially reshaped the microbial composition. Altogether, our findings support the role of regular exercise in maintaining the stability of the gut microbiota in elderly individuals and reveal that regular exercise benefits OE individuals.

## CONFLICT OF INTEREST

None declared.

## AUTHOR CONTRIBUTIONS

**Qiwei Zhu:** Data curation (lead). **Shangfei Jiang:** Software (lead). **Guankui Du:** Conceptualization (lead); Formal analysis (lead); Funding acquisition (lead); Investigation (lead); Methodology (lead); Project administration (lead); Resources (lead); Writing – original draft (lead); Writing – review and editing (lead).

## ETHICS STATEMENT

None required.

## Data Availability

All data used for this study are available at https://www.ebi.ac.uk/ena/browser/view/PRJEB11419

## APPENDIX 1

**TABLE A1:** The relative abundances of 129 pathways were significantly changed in OE individuals compared with the NW groups

Pathway	NW relative frequency (%) mean ± *SD*	OW relative frequency (%) mean ± *SD*	*p*‐Values
PWY‐5971	0.1931 ± 0.1349	0.2708 ± 0.1734	5.45E−11
PENTOSE‐P‐PWY	0.4021 ± 0.1285	0.3532 ± 0.1140	4.87E−08
FASYN‐INITIAL‐PWY	0.1628 ± 0.1234	0.2150 ± 0.1393	1.23E−07
PWY‐7664	0.2106 ± 0.1528	0.2746 ± 0.1695	1.24E−07
GLCMANNANAUT‐PWY	0.2290 ± 0.0710	0.2037 ± 0.0604	1.62E−07
PWYG‐321	0.2146 ± 0.1528	0.2771 ± 0.1663	1.64E−07
PWY‐6282	0.1902 ± 0.1449	0.2501 ± 0.1612	1.85E−07
PWY‐5989	0.1918 ± 0.1481	0.2520 ± 0.1639	2.57E−07
POLYAMINSYN3‐PWY	0.0616 ± 0.0379	0.0759 ± 0.0372	3.11E−07
PWY0‐862	0.1926 ± 0.1447	0.2505 ± 0.1594	3.67E−07
PWY‐5505	0.5451 ± 0.1688	0.6013 ± 0.1448	1.11E−06
PWY‐7007	0.0008 ± 0.0063	0.0035 ± 0.0079	1.13E−06
PWY‐5484	0.6491 ± 0.1140	0.6094 ± 0.1114	1.98E−06
GLUCARGALACTSUPER‐PWY	0.0222 ± 0.0255	0.0149 ± 0.0179	4.96E−06
GALACTARDEG‐PWY	0.0222 ± 0.0255	0.0149 ± 0.0179	4.96E−06
PWY‐6071	0.0035 ± 0.0105	0.0077 ± 0.0138	5.75E−06
REDCITCYC	0.1191 ± 0.1076	0.0889 ± 0.0776	9.24E−06
PWY‐7332	0.0137 ± 0.0213	0.0083 ± 0.0128	1.67E−05
GLYCOLYSIS	0.7464 ± 0.0847	0.7200 ± 0.0824	1.90E−05
PWY‐6901	0.4311 ± 0.1174	0.3968 ± 0.1061	3.05E−05
PWY‐7446	0.0082 ± 0.0151	0.0045 ± 0.0098	3.95E−05
PWY‐7220	0.6884 ± 0.1044	0.6587 ± 0.0970	6.21E−05
PWY‐7222	0.6884 ± 0.1044	0.6587 ± 0.0970	6.21E−05
METHGLYUT‐PWY	0.0120 ± 0.0211	0.0069 ± 0.0141	6.84E−05
PWY‐6519	0.2150 ± 0.1206	0.2500 ± 0.1223	9.87E−05
P108‐PWY	0.4574 ± 0.1624	0.4143 ± 0.1473	0.0002
PWY‐5676	0.1208 ± 0.0744	0.1039 ± 0.0573	0.0005
PWY‐5180	0.0253 ± 0.0326	0.0178 ± 0.0260	0.0005
PWY‐5182	0.0253 ± 0.0326	0.0178 ± 0.0260	0.0005
GLUCUROCAT‐PWY	0.2137 ± 0.0745	0.2324 ± 0.0707	0.0005
PWY‐6471	0.1483 ± 0.1003	0.1240 ± 0.0903	0.0005
PWY0‐41	0.0124 ± 0.0189	0.0085 ± 0.0122	0.0006
GLUCARDEG‐PWY	0.0246 ± 0.0256	0.0189 ± 0.0192	0.0006
BIOTIN‐BIOSYNTHESIS‐PWY	0.2195 ± 0.1112	0.2471 ± 0.1076	0.0006
TCA	0.5675 ± 0.1223	0.5385 ± 0.1119	0.0007
PWY‐6478	0.0414 ± 0.0411	0.0517 ± 0.0422	0.0008
PWY‐7090	0.0022 ± 0.0032	0.0016 ± 0.0018	0.0008
GALACT‐GLUCUROCAT‐PWY	0.2319 ± 0.0727	0.2490 ± 0.0679	0.0009
PWY‐5022	0.0369 ± 0.0366	0.0292 ± 0.0276	0.0010
PWY‐6690	0.0101 ± 0.0171	0.0067 ± 0.0118	0.0013
HCAMHPDEG‐PWY	0.0101 ± 0.0171	0.0067 ± 0.0118	0.0013
PWY0‐321	0.0051 ± 0.0157	0.0090 ± 0.0175	0.0014
PYRIDNUCSAL‐PWY	0.6468 ± 0.0809	0.6636 ± 0.0631	0.0014
PWY‐7254	0.0876 ± 0.0899	0.0694 ± 0.0668	0.0015
GLYOXYLATE‐BYPASS	0.0492 ± 0.0623	0.0369 ± 0.0434	0.0015
PWY‐5677	0.0271 ± 0.0244	0.0225 ± 0.0166	0.0024
PWY‐7663	0.9422 ± 0.0817	0.9574 ± 0.0553	0.0025
PWY‐6588	0.1974 ± 0.1010	0.1774 ± 0.0814	0.0027
RIBOSYN2‐PWY	0.6539 ± 0.0671	0.6670 ± 0.0524	0.0027
KETOGLUCONMET‐PWY	0.0134 ± 0.0231	0.0090 ± 0.0166	0.0028
P42‐PWY	0.7444 ± 0.1884	0.7062 ± 0.1643	0.0031
PWY0‐1533	0.0230 ± 0.0272	0.0178 ± 0.0211	0.0032
PWY0‐1296	0.4445 ± 0.1115	0.4673 ± 0.0999	0.0032
ANAEROFRUCAT‐PWY	0.6227 ± 0.0880	0.6047 ± 0.0804	0.0034
PWY‐6969	0.6513 ± 0.1314	0.6249 ± 0.1149	0.0035
PWY‐6126	0.7834 ± 0.0636	0.7700 ± 0.0612	0.0036
PWY‐5913	0.4997 ± 0.1519	0.4689 ± 0.1391	0.0040
PWY‐7228	0.4718 ± 0.1020	0.4510 ± 0.0956	0.0042
P341‐PWY	0.0014 ± 0.0052	0.0006 ± 0.0025	0.0051
PWY‐7197	0.3681 ± 0.0967	0.3488 ± 0.0916	0.0052
PWY‐6125	0.5017 ± 0.0964	0.4826 ± 0.0911	0.0054
PWY‐5747	0.0136 ± 0.0187	0.0103 ± 0.0137	0.0060
PWY‐5667	0.8401 ± 0.0899	0.8569 ± 0.0787	0.0065
PWY0‐1319	0.8401 ± 0.0899	0.8569 ± 0.0787	0.0065
P122‐PWY	0.1046 ± 0.0615	0.0939 ± 0.0459	0.0065
PWY‐5705	0.0176 ± 0.0170	0.0146 ± 0.0129	0.0069
PWY‐5154	0.3929 ± 0.1133	0.3715 ± 0.1030	0.0070
GLYCOCAT‐PWY	0.8738 ± 0.1187	0.8961 ± 0.1077	0.0074
PWY‐841	0.5890 ± 0.0899	0.5716 ± 0.0869	0.0074
PWY‐7200	0.3806 ± 0.0613	0.3691 ± 0.0566	0.0075
PWY‐6163	0.7938 ± 0.0703	0.8064 ± 0.0595	0.0077
PWY‐5347	0.3726 ± 0.1086	0.3926 ± 0.0957	0.0078
PWY‐621	0.5584 ± 0.1486	0.5841 ± 0.1179	0.0083
PWY0‐42	0.0110 ± 0.0177	0.0082 ± 0.0117	0.0083
PWY0‐1277	0.0164 ± 0.0261	0.0119 ± 0.0199	0.0083
PWY‐7184	0.3518 ± 0.0778	0.3376 ± 0.0701	0.0088
PWY0‐845	0.2685 ± 0.1161	0.2478 ± 0.1005	0.0092
PWY‐5973	0.8943 ± 0.0760	0.9063 ± 0.0516	0.0099
GLYCOGENSYNTH‐PWY	0.8051 ± 0.1419	0.8306 ± 0.1305	0.0109
TCA‐GLYOX‐BYPASS	0.0564 ± 0.0703	0.0451 ± 0.0517	0.0112
PWY‐5532	0.0010 ± 0.0020	0.0007 ± 0.0012	0.0114
GOLPDLCAT‐PWY	0.0068 ± 0.0127	0.0094 ± 0.0152	0.0125
PYRIDOXSYN‐PWY	0.2254 ± 0.1127	0.2057 ± 0.1032	0.0129
DENOVOPURINE2‐PWY	0.5938 ± 0.0826	0.5789 ± 0.0821	0.0137
PWY‐7237	0.0543 ± 0.0561	0.0630 ± 0.0411	0.0144
PWY‐4984	0.1049 ± 0.0787	0.0935 ± 0.0530	0.0178
PWY‐6165	0.0016 ± 0.0035	0.0025 ± 0.0058	0.0204
PWY‐7196	0.5429 ± 0.1101	0.5243 ± 0.1087	0.0214
ARO‐PWY	0.7795 ± 0.0856	0.7929 ± 0.0745	0.0214
FAO‐PWY	0.0545 ± 0.0568	0.0460 ± 0.0451	0.0223
PHOSLIPSYN‐PWY	0.7803 ± 0.0894	0.7941 ± 0.0763	0.0223
PWY0‐166	0.4147 ± 0.0681	0.4042 ± 0.0579	0.0225
P164‐PWY	0.1413 ± 0.0669	0.1315 ± 0.0506	0.0225
PWY‐7199	0.5239 ± 0.0738	0.5119 ± 0.0700	0.0227
PWY0‐781	0.2635 ± 0.0963	0.2488 ± 0.0801	0.0228
PWY‐5659	0.5976 ± 0.0871	0.5835 ± 0.0816	0.0232
CODH‐PWY	0.0179 ± 0.0216	0.0213 ± 0.0190	0.0234
P124‐PWY	0.1339 ± 0.0888	0.1210 ± 0.0676	0.0238
GLYCOL‐GLYOXDEG‐PWY	0.0291 ± 0.0321	0.0245 ± 0.0239	0.0245
PWY‐5415	0.0065 ± 0.0148	0.0045 ± 0.0104	0.0254
PWY0‐162	0.5985 ± 0.0980	0.5828 ± 0.0940	0.0255
RUMP‐PWY	0.0667 ± 0.0562	0.0588 ± 0.0413	0.0267
PWY‐5304	0.1095 ± 0.0954	0.1243 ± 0.0881	0.0276
COMPLETE‐ARO‐PWY	0.8166 ± 0.0871	0.8297 ± 0.0758	0.0278
HISTSYN‐PWY	0.7138 ± 0.0779	0.7017 ± 0.0732	0.0290
PWY‐7187	0.4234 ± 0.0635	0.4139 ± 0.0558	0.0299
P163‐PWY	0.0352 ± 0.0357	0.0303 ± 0.0257	0.0301
PWY‐6185	0.0024 ± 0.0074	0.0038 ± 0.0095	0.0301
PWY‐5910	0.0185 ± 0.0400	0.0131 ± 0.0289	0.0303
PWY‐6151	0.4874 ± 0.1112	0.5037 ± 0.0971	0.0319
SER‐GLYSYN‐PWY	0.7444 ± 0.0713	0.7552 ± 0.0664	0.0328
PWY‐922	0.0135 ± 0.0309	0.0095 ± 0.0217	0.0334
PWY‐5695	0.6651 ± 0.1110	0.6481 ± 0.1065	0.0338
PWY‐7013	0.0434 ± 0.0496	0.0369 ± 0.0356	0.0354
PWY‐6608	0.2527 ± 0.1048	0.2378 ± 0.0902	0.0365
PWY‐6470	0.0213 ± 0.0356	0.0165 ± 0.0271	0.0380
PWY‐6182	0.0024 ± 0.0074	0.0037 ± 0.0093	0.0381
PWY‐5198	0.0005 ± 0.0018	0.0013 ± 0.0068	0.0387
GLUCOSE1PMETAB‐PWY	0.0470 ± 0.0659	0.0393 ± 0.0342	0.0389
ARGSYNBSUB‐PWY	0.6160 ± 0.1288	0.6344 ± 0.1157	0.0399
TEICHOICACID‐PWY	0.0879 ± 0.0496	0.0950 ± 0.0452	0.0412
GLYCOLYSIS‐TCA‐GLYOX‐BYPASS	0.0819 ± 0.0870	0.0706 ± 0.0667	0.0441
CATECHOL‐ORTHO‐CLEAVAGE‐PWY	0.0025 ± 0.0069	0.0038 ± 0.0105	0.0449
PWY‐3781	0.1989 ± 0.1952	0.1734 ± 0.1545	0.0452
THREOCAT‐PWY	0.0054 ± 0.0166	0.0080 ± 0.0182	0.0458
PWY‐5417	0.0027 ± 0.0076	0.0041 ± 0.0111	0.0468
PWY‐5431	0.0027 ± 0.0076	0.0041 ± 0.0111	0.0468
PWY‐7560	0.7715 ± 0.0707	0.7811 ± 0.0632	0.0497
NONMEVIPP‐PWY	0.7715 ± 0.0707	0.7811 ± 0.0632	0.0497

## APPENDIX 2

**TABLE A2:** The relative abundances of 24 pathways were significantly changed in DROE compared with NROE

Pathway	NROE relative frequency (%) mean ± *SD*	DROE relative frequency (%) mean ± *SD*	p‐values
PWY‐7090	0.0015 ± 0.0016	0.0020 ± 0.0029	0.0052
PWY‐7332	0.0084 ± 0.0138	0.0120 ± 0.0192	0.0077
PWY‐5484	0.6120 ± 0.1064	0.6382 ± 0.1169	0.0077
GLYCOLYSIS	0.7214 ± 0.0761	0.7392 ± 0.0873	0.0121
PWY‐841	0.5670 ± 0.0867	0.5853 ± 0.0904	0.0201
PWY‐7228	0.4474 ± 0.0969	0.4669 ± 0.1017	0.0274
DENOVOPURINE2‐PWY	0.5743 ± 0.0830	0.5907 ± 0.0835	0.0289
P108‐PWY	0.4183 ± 0.1360	0.4460 ± 0.1627	0.0305
PWY‐6125	0.4791 ± 0.0936	0.4972 ± 0.0959	0.0335
PWY‐7184	0.3343 ± 0.0715	0.3481 ± 0.0773	0.0351
GLUCUROCAT‐PWY	0.2332 ± 0.0724	0.2194 ± 0.0764	0.0367
PWY‐6519	0.2480 ± 0.1223	0.2251 ± 0.1235	0.0379
PWY‐6353	0.2724 ± 0.0803	0.2575 ± 0.0765	0.0380
PWY‐4984	0.0908 ± 0.0592	0.1024 ± 0.0713	0.0381
PENTOSE‐P‐PWY	0.3659 ± 0.1145	0.3875 ± 0.1281	0.0415
PWY‐7197	0.3461 ± 0.0930	0.3633 ± 0.0969	0.0423
PWY‐7200	0.3666 ± 0.0601	0.3776 ± 0.0599	0.0433
PWY‐5505	0.5909 ± 0.1517	0.5628 ± 0.1648	0.0437
GALACT‐GLUCUROCAT‐PWY	0.2503 ± 0.0707	0.2374 ± 0.0741	0.0441
PWY0‐162	0.5776 ± 0.0972	0.5953 ± 0.0975	0.0448
PWY0‐166	0.4008 ± 0.0591	0.4118 ± 0.0667	0.0456
P125‐PWY	0.0074 ± 0.0160	0.0112 ± 0.0365	0.0471
PWY0‐1296	0.4674 ± 0.1002	0.4492 ± 0.1098	0.0483
PWY‐7196	0.5194 ± 0.1089	0.5388 ± 0.1110	0.0491
